# Why students do not turn on their video cameras during online classes and an equitable and inclusive plan to encourage them to do so

**DOI:** 10.1002/ece3.7123

**Published:** 2021-01-10

**Authors:** Frank R. Castelli, Mark A. Sarvary

**Affiliations:** ^1^ Investigative Biology Teaching Laboratories Department of Neurobiology and Behavior Cornell University Ithaca NY USA

**Keywords:** distance learning, remote instruction, synchronous teaching, video cameras, videoconferencing, Zoom

## Abstract

Enrollment in courses taught remotely in higher education has been on the rise, with a recent surge in response to a global pandemic. While adapting this form of teaching, instructors familiar with traditional face‐to‐face methods are now met with a new set of challenges, including students not turning on their cameras during synchronous class meetings held via videoconferencing. After transitioning to emergency remote instruction in response to the COVID‐19 pandemic, our introductory biology course shifted all in‐person laboratory sections into synchronous class meetings held via the Zoom videoconferencing program. Out of consideration for students, we established a policy that video camera use during class was optional, but encouraged. However, by the end of the semester, several of our instructors and students reported lower than desired camera use that diminished the educational experience. We surveyed students to better understand why they did not turn on their cameras. We confirmed several predicted reasons including the most frequently reported: being concerned about personal appearance. Other reasons included being concerned about other people and the physical location being seen in the background and having a weak internet connection, all of which our exploratory analyses suggest may disproportionately influence underrepresented minorities. Additionally, some students revealed to us that social norms also play a role in camera use. This information was used to develop strategies to encourage—without requiring—camera use while promoting equity and inclusion. Broadly, these strategies are to not require camera use, explicitly encourage usage while establishing norms, address potential distractions, engage students with active learning, and understand your students’ challenges through surveys. While the demographics and needs of students vary by course and institution, our recommendations will likely be directly helpful to many instructors and also serve as a model for gathering data to develop strategies more tailored for other student populations.

## Graphical abstract

Students were asked why they chose not to turn on their cameras during synchronous class meetings held via Zoom. Their responses influenced a strategy for encouraging them to do so.

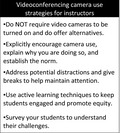

## 1 INTRODUCTION

Student enrollment in distance education courses in postsecondary institutions has been on the rise (NCES, 2020; Palvia et al., 2018). Some forms of distance learning utilize advances in technology that allow for synchronous class meetings over the internet using videoconferencing software (Al‐Samarraie, 2019). Moreover, the ongoing COVID‐19 pandemic has led to a widespread need for instruction to move online and for instructors to hold synchronous class meetings using videoconferencing software to maintain social distancing and prevent the spread of infection (The Chronicle of Higher Education, 2020; UNESCO, 2020; Yuan, 2020). Many educators now find themselves teaching remotely for the first time and facing a new set of challenges (e.g., Reich et al., 2020). One such challenge in the world of remote instruction, and the focus of this study, is not being able to see students during synchronous class meetings held via videoconferencing software, because students do not have their video cameras turned on.

Midway in the spring semester of 2020, like at many other institutions in the United States, courses at Cornell University made an emergency shift to remote instruction due to the COVID‐19 pandemic (Cornell University, 2020; Milman, 2020), and our introductory biology laboratory course, covering topics in ecology and evolution, was among them. We desired to continue the course with synchronous laboratory section meetings due to the many educational benefits for students of synchronous remote learning compared to asynchronous.

Benefits of synchronous remote learning include the following: opportunities for higher interactivity and engagement among students, timely and constructive feedback, and real‐time collaborative learning (Racheva, 2018). Additionally, synchronous learning helps to build a stronger sense of community that fosters interactions, discussions, and the sharing of ideas (Lin & Gao, 2020), something we valued to maintain after transitioning but is also important in future semesters as new cohorts of students begin classes remotely. Another benefit of synchronous learning is that the increased social interaction that comes with it compared to asynchronous learning is strongly related to greater remote learning enjoyment, improved effectiveness of remote learning, and a higher likelihood of enrolling in another online class (Muilenburg & Berge, 2005). Student performance in synchronous remote courses also has the potential to be similar to performance in face‐to‐face versions of the same course (e.g., Francescucci & Rohani, 2019). Lastly, interactions from synchronous instruction may help to counter the effects of social distancing policies that can increase feelings of loneliness and result in negative cognitive (Cacioppo & Hawkley, 2009) and other health consequences (Aleman & Sommer, 2020).

Despite our desires to teach synchronously while remote for the rest of the semester, we, and the rest of the course staff, endeavor to practice inclusive pedagogy, which includes having a mind‐set that values course design and policies that do not exclude students due to inequities (Gannon, 2018). Hence, we were initially concerned about the “digital divide,” in that many of our students might not have access to a reliable internet connection or to a computer with a working webcam and microphone (Cullen, 2001). To find out if this was the case, we surveyed our students during a pause in classes before remote instruction began. Fortunately, only a small percentage of students indicated these problems (webcam: 2%; internet: 5%; *N* = 301). Therefore, we felt it was not unreasonable to resume instruction for our laboratory sections in the form of live synchronous class meetings held via Zoom (Zoom Video Communications), with video recordings of meetings and asynchronous means for participation for those who could not attend synchronously. Students without reliable access to the necessary technologies were also referred to university resources put in place in response to the pandemic.

After deciding that we would hold synchronous class meetings, the next question became whether we should mandate that students turn on their video cameras during class. The supervising staff agreed that the best student‐centered policy would be to *not require* students to use cameras because they may not be comfortable doing so, particularly if they do not have access to a private space or are embarrassed of their home environment (Costa, 2020). Such concerns have been expressed by other educators (Reich et al., 2020). The COVID‐19 pandemic has already increased college student anxiety and depression (Huckins et al., 2020), and a mandate for camera use may add to that trauma (Costa, 2020). Furthermore, students of some populations may be disproportionately affected by stress from the pandemic (McGinty et al., 2020). For example, national surveys of adults in the United States show that measures of symptoms of psychological distress increased from about 4% to 14% from 2018 to April 2020 with the highest increases in adults aged 18–29 (4%–24%), adults with household incomes of less than $35,000/year (8%–19%), and Hispanic adults (4%–18%) (McGinty et al., 2020). Thus, the negative effects of mandating that cameras be kept on may also be disproportional to some student populations, such as underrepresented minorities in STEM (Science, Technology, Engineering, and Mathematics), and contribute to already high attrition rates (PCAST, 2012).

There are several reasons that students having cameras on may be beneficial for teaching and learning, justifying our encouragement of their use, and we review some of them here. Perhaps the most obvious benefit is the ability to communicate with nonverbal cues. Instructors benefit from receiving nonverbal cues from their students such as smiles, frowns, head nods, looks of confusion, and looks of boredom, so that they can evaluate their teaching in real time and adjust accordingly to improve student learning (Miller, 1988; Mottet & Richmond, 2002). Instructors that perceive a higher amount of nonverbal responsiveness also rate themselves as having been more effective (Mottet, 2000). Students similarly benefit from being able to see other students when collaboratively learning. Students with video in addition to audio were more able than students with only audio to tell how their other study group members were reacting to things they said while remote learning (Olson et al., 1995). The inclusion of video has also been suggested as a way to address the fact that students in remote classes without video reported that a lack of nonverbal communication “reduced their educational experience” (McBrien et al., 2009).

In addition to improving instructor effectiveness, being able to see students while teaching makes for a more positive affective experience. For example, instructors that perceived a higher amount of nonverbal responsiveness reported higher satisfaction and a higher preference for wanting to teach in a remote video class (Mottet, 2000). Additionally, instructors of our course expressed displeasure with a general feeling of “talking to yourself” that occurs when students do not have their cameras on. This is consistent with feedback from remote meeting participants who preferred videoconferencing over audio‐only conferencing because the former had the advantage of “not talking into a void” (O'Conaill et al., 1993). If instructors have a negative experience, they may do a poorer job teaching, and poor teaching may lead students to switch out of science, math, and engineering majors (Seymour & Hewitt, 1997).

Students having their cameras on also helps to build instructor‐student and student–student relationships. Instructors that perceived a higher amount of nonverbal responsiveness while teaching remotely with video reported instructor–student interpersonal relationships that were warmer, closer, and more comfortable (Mottet, 2000). The majority of students of a virtual classroom indicated that using videoconferencing helped build trust and rapport with other students and helped them to develop a sense of identification with others in their group, commenting that being able to hear and see each other in real time helped construct a “more complete picture” of their peers (Falloon, 2011). Moreover, building stronger relationships via videoconferencing likely helps fend off loneliness that may come with remote learning and social distancing, as videoconferencing has been shown to decrease feelings of loneliness in nursing home residents (Tsai et al., 2010).

Nevertheless, by the end of the semester, many students had often chosen to leave their cameras off, as reported by our frustrated instructors. This attrition with camera use in online classes has been reported elsewhere (Tonsmann, 2014). Given the potential benefits of students having their cameras on and the costs of having their cameras off, we were wondering what strategies we could use to encourage students to voluntarily turn on their cameras during laboratory sections. To develop strategies informed by data, we asked our students in an end‐of‐semester survey about why they might have chosen to not turn on their video cameras during class. In this paper, we present the results of that survey and the strategies we developed based on those results in combination with successful experiences of some of our instructors, conversations with colleagues, suggestions from articles and social media, and a review of the literature. We plan to try these strategies going forward as the need for remote instruction continues for us as well as many instructors across the globe during the COVID‐19 pandemic (UNESCO, 2020). While the demographics and needs of students vary by course and institution, we believe that these strategies are likely to be helpful to many instructors directly, or at least, serve as a model for gathering data to develop strategies more tailored for their students.

## 2 METHODS AND MATERIALS

### 2.1 Course description

The students surveyed were undergraduates enrolled in the Investigative Biology Laboratory course at Cornell University (Ithaca, NY, USA), a PhD‐granting institution, in the spring semester of 2020. This course is required for majors in the biological sciences and uses an inquiry‐based approach to teaching the scientific method within two main topics: the evolution of antibiotic resistance in bacteria and the ecological impact of limiting nutrients on algal growth. Before the COVID‐19 pandemic, this course was taught with one 50‐min lecture and one 3‐hr laboratory section per week. After the switch to emergency remote instruction (Cornell University, 2020; Milman, 2020), lectures were prerecorded for asynchronous viewing and sections were held synchronous via Zoom (Zoom Video Communications) with recordings available for asynchronous viewing. We decided on this format after surveying our students before remote instruction began in order to assess the challenges they would be facing once they left campus, including differences in time zones, distractions from family obligations, and access to technology. Each of 12 graduate student laboratory instructors led two sections, and most were assisted by one undergraduate teaching assistant. Section enrollment was capped at 18 students. During synchronous laboratory sections, instructors gave short presentations while screen‐sharing a slideshow and incorporating active learning techniques such as polling (Sarvary & Gifford, 2017) and think‐pair‐share (Tanner, 2013). For several weeks, a portion of each class meeting was devoted to students meeting with their long‐term groups to work on data analysis and a poster presentation using the breakout room feature of Zoom. Instructors could move between breakout rooms to help students. Students also presented their group posters to the rest of the class and responded to questions during the last synchronous class meeting.

### 2.2 Student sample

Total enrollment for spring 2020 was 312 students. Ninety‐one percent of enrolled students took the end‐of‐semester survey (*N* = 283), and seven respondents were excluded from analysis because they either did not attend any live synchronous remote class meetings or skipped all the relevant questions, resulting in a final sample of 88% (*N* = 276) of enrolled students.

Students were assigned demographic categories based on self‐reporting and had the option to skip demographic survey questions or indicate that they choose not to disclose. Results were broken down by gender based on male and female, as only one respondent identified as nonbinary (*N* = 1). Students were asked about their race and ethnicity (“How would you describe yourself? Check all that apply.”), and their responses were used to assign them an underrepresented minority (URM) in science and engineering or a non‐URM. URMs were defined as three racial or ethnic minority groups: blacks or African Americans; Hispanics, Latinx, or Spanish origin; and American Indians or Alaska Natives, or a mix including one of these groups (NSF, 2019). Non‐URMs included: whites; Asians; Native Hawaiians or other Pacific Islanders. URM status was unknown for 23 students. Freshman or non‐Freshman (Sophomore, Junior, or Senior) status was unknown for ten students. Non‐Freshman were combined due to relatively small sample sizes (Freshmen, *N* = 215; Sophomores, *N* = 32; Juniors, *N* = 14; Seniors, *N* = 5).

### 2.3 End‐of‐semester survey

Students were administered an end‐of‐semester survey designed to collect feedback on teaching, course design, and student experiences within the course. The survey was administered with the Qualtrics online survey tool (Qualtrics) during their last synchronous section meeting of the semester. Laboratory instructors were directed to email any absent student after class with a link to the survey and a deadline of a few days. The survey was anonymous, most questions were able to be skipped, and taking it was completely voluntary. No credit was awarded for taking the survey.

The main survey question analyzed for the current study asked, “If you ever left your video off during the live Zoom lab meetings, why did you leave it off? (check all that apply).” Students could select up to 12 reasons we hypothesized a priori or select “Not Applicable – I always had my camera on.” When selecting “Other,” students also had the option of typing a reason not listed. In order to be included in analysis, students had to indicate that they attend at least some of the synchronous class meetings (“Have you attended any of the Zoom lab meetings as they occurred in real time?”). Additionally, students indirectly offered insight into the phenomenon of leaving cameras off during class when responding to other survey questions that solicited feedback about the course, the emergency switch to remote instruction, and the performance of laboratory instructors.

### 2.4 Statistical analyses

For each of the 13 possible answer choices to the question: “If you ever left your video off during the live Zoom lab meetings, why did you leave it off? (check all that apply),” pairwise differences between demographic categories (URM vs. non‐URM, male vs. female, and Freshman vs. non‐Freshman) were analyzed with two‐tailed Fisher's exact tests using the GraphPad QuickCalcs website (GraphPad, 2020). The *α* level for this study was set at 0.05. We chose not to correct for multiple comparisons by adjusting the significance threshold, because we consider these analyses to be exploratory, with significant results requiring subsequent study for confirmation (Bender & Lange, 2001).

## 3 RESULTS

The vast majority of students (*N* = 249, 90%) had their video cameras off at least some of the time during remote synchronous class meetings held via Zoom. Students indicated several reasons for doing so. Table 1 summarizes the percentages of students selecting each reason from a provided list, along with break downs by demographic categories. The most frequently selected reason overall and across all demographic categories was being concerned about appearance (*N* = 113, 41% of students). The next most frequently selected reason, a concern about other people being seen in the background, was selected by substantially fewer students overall, but was still quite common (*N* = 73, 26%). This reason was selected more frequently than the related reason of not wanting their physical location to be seen in the background. Of relatively moderate frequency were concerns about distracting their classmates or instructor. Not wanting to be seen walking away from the computer, not paying attention, or doing other things while at the computer were relatively infrequently selected overall and across demographic break downs. As for reasons related to technology, a very small number of students (*N* = 6, 2% of students) reported that their webcam was not working, but a much larger number (*N* = 61, 22%) reported having a weak internet connection.

**TABLE 1:** Reasons undergraduate students gave for not turning on their video cameras during synchronous online class meetings in a survey given in an introductory biology laboratory course at a four‐year PhD‐granting institution at the end of the spring 2020 semester

Reasons for not turning on camera	All students	URM	Non‐URM	Male	Female	Freshman	Non‐Freshman
I was concerned about my appearance	41%	45%	38%	36%	43%	38%	49%
I was concerned about other people being seen behind me	26%	38%	24%	20%	32%	28%	22%
My internet connection was weak	22%	32%	20%	15%	27%	23%	18%
Other [with space to enter text]	19%	15%	20%	18%	20%	18%	25%
I felt like everyone was looking at me the whole time	17%	20%	17%	16%	18%	18%	14%
I was concerned about my physical location being seen behind me	17%	26%	13%	9%	21%	16%	20%
I was concerned about distracting my classmates	17%	12%	21%	16%	19%	17%	20%
I was concerned about distracting my lab instructor	12%	14%	13%	14%	12%	13%	12%
Not Applicable ‐ I always had my camera on	10%	8%	11%	12%	9%	10%	10%
I didn't want to be seen not paying attention	8%	8%	7%	9%	7%	7%	10%
I didn't want to be seen walking away from my computer	7%	11%	6%	8%	7%	7%	10%
I didn't want to be seen doing other things on my computer	7%	8%	7%	10%	5%	7%	10%
My webcam was not working.	2%	3%	2%	3%	2%	3%	0%
Number of students	276	66	187	99	164	215	51

Note

Each student that indicated they did not always have their camera on could select more than one reason. Reasons are broken down by underrepresented minorities (URM) in science and engineering status, gender, and Freshman or non‐Freshman (sophomores, juniors, and seniors) and are sorted in descending order according to all student respondents combined.

URMs more than non‐URMs selected “I was concerned about other people being seen behind me” (URM: *N* = 25/66; non‐URM: *N* = 45/187; *p* = 0.0376) and “I was concerned about my physical location being seen behind me” (URM: *N* = 17/66; non‐URM: *N* = 24/187; *p* = 0.0194). For the same two reasons, females more than males selected “I was concerned about other people being seen behind me” (Male: *N* = 20/99; Female: *N* = 52/164; *p* = 0.0465) and “I was concerned about my physical location being seen behind me” (Male: *N* = 9/99; Female: *N* = 35/164; *p* = 0.0104). However, URMs were not more likely to be females (Female URM: *N* = 45/161 [28%], Male URM: *N* = 21/91 [23%]; *p* = 0.4569). For “My internet connection was weak,” females were significantly more likely than males to select this reason (Male: *N* = 15/99; Female: *N* = 44/164; *p* = 0.0326) and URMS only tended to select this reason more than non‐URMs (URM: *N* = 21/66; non‐URM: *N* = 37/187; *p* = 0.0603).

A sizeable number of students selected “Other” from the list of provided reasons (*N* = 53, 19%) and could then type in a reason. One student did not state a reason, three gave two reasons each and the rest each gave only one. The open‐ended responses were emergently coded and placed into 15 categories summarized in Table 2. By far, the most common category was “It was the norm,” with just over half of the typed reasons falling in this category (*N* = 28, 53%). Representative statements from this category include: “Everyone else had their camera off,” and, “[it] felt awkward having it on if no one else did.” Although concern about appearance was an option to select in the provided list of reasons, four students used the “Other” option to elaborate, mentioning messy hair, wearing pajamas, not having showered, and even crying (unrelated to the course). Examples from “Not feeling a need to have the camera on” include: “I did not see a need to turn on the video…,” and “…I felt like it wasn't necessary for my learning.” An example of “Not wanting the camera to be on or to be seen, in general” is: “I just didn't want my video on.”

**TABLE 2:** Additional reasons undergraduate students of a biology class gave for not turning on their video cameras during synchronous online class meetings

"Other" reasons given sorted into categories	Percent	Count
It was the norm	52.8%	28
Being concerned about appearance (elaborations for this concern)	7.5%	4
Wanting to improve the internet connection/streaming smoothness	7.5%	4
Not feeling a need to have the camera on	7.5%	4
Not wanting to divert attention from the instructor	5.7%	3
Not wanting the camera to be on or to be seen, in general	5.7%	3
Not wanting to be seen eating	3.8%	2
Not wanting to be seen in bed	1.9%	1
Being distracted by seeing one's own video feed	1.9%	1
Being more comfortable with the camera off	1.9%	1
Having to talk to family members	1.9%	1
Leaving the computer to use the bathroom	1.9%	1
Not having a webcam	1.9%	1
The default setting of the Zoom program was to join without video	1.9%	1
No “other” reason given	1.9%	1

Note

Reasons were categorized from typed responses after choosing “Other” from a provided list of reasons. Percentages are of those respondents choosing “Other.”

Students also provided additional feedback regarding video cameras being on or off during synchronous class meetings when providing open‐ended answers to other survey questions. Select quotes are drawn into the discussion that follows.

## 4 DISCUSSION

### 4.1 Overview

By surveying our students to ask why they did not always turn on their video cameras during synchronous class meetings, we gained valuable information to help us to develop strategies for encouraging students to turn on their video during class. With this information, in combination with feedback from our course instructors as well as conversations with colleagues, published research, and suggestions from articles and social media, we developed the following strategies for instructors to implement in remote instruction moving forward, summarized in Table 3. In developing these strategies, we took a student‐centered approach, being mindful of equity, inclusion, and the diversity of our students and the situations they find themselves in while learning remotely. We encourage any instructor to gather information about their own students to better tailor these strategies.

**TABLE 3:** Our proposed videoconferencing strategies for instructors to encourage, but not require, students to turn on their cameras during synchronous class meetings

Videoconferencing camera use strategies for instructors
Do NOT require video cameras to be turned on and do offer alternatives
Explicitly encourage camera use, explain why you are doing so, and establish the norm
Address potential distractions and give breaks to help maintain attention
Use active learning techniques to keep students engaged and promote equity
Survey your students to understand their challenges

### 4.2 Do not require video cameras to be turned on and do offer alternatives

By taking a student‐centered approach, we make pedagogical decisions based on what is best for the student's learning, not the instructor's teaching per se. While some instructors may feel a strong need to mandate that students turn on their video cameras during class, we strongly advise against this. While we surveyed students to find broad reasons why they chose not to turn on their cameras, there are many more specific and very sensitive, reasons for their not doing so. One of our surveyed students strongly captured this point when asked about any challenges they have faced with the transition to online learning: “I would like to mention that no one should assume the living conditions of students when not on campus. Some students live in some of the worst conditions possible.” This concern is consistent with those of educators reported elsewhere (Reich et al., 2020). For example, one teacher told researchers that their students of working class families do not want to turn on their cameras because they do not want their wealthy peers to see inside their homes and another said they have students that lack private spaces and end up connecting to Zoom from inside a closet (Reich et al., 2020).

College student anxiety and depression have already been increased by the COVID‐19 pandemic (Huckins et al., 2020), and a mandate for camera use may add to that trauma (Costa, 2020). Furthermore, psychological distress due to the pandemic has disproportionately affected adults 18–29 years old, adults from low income homes, and Hispanic adults (McGinty et al., 2020). Thus, the negative effects of mandating that cameras be kept on may also be disproportional to some student populations, such as underrepresented minorities in STEM, and contribute to already high attrition rates (PCAST, 2012).

We will offer alternative means for students to participate and communicate with the instructor and the rest of the class, such as polling (Sarvary & Gifford, 2017), discussion boards (Suler, 2004), shared documents (e.g., Google Docs; Perron & Sellers, 2011), and cooperative annotations (Zhu et al., 2020). Additionally, our instructors have had great success using the chat feature within Zoom. Some of them reported that individuals that did not often speak when class was in‐person were suddenly more communicative when using the chat feature online. We also found that our undergraduate teaching assistants were helpful facilitating student participation in several ways including monitoring the chat window during class, creating polls, and managing discussion boards (Asgari & Sarvary, 2020). Having multiple avenues for participation should improve equity and likely benefit all students, not just those that do not turn on their camera. Our students appreciated this strategy. When asked, “What are some of the positive aspects about how your lab instructor taught specifically during the online Zoom meetings?” one surveyed student wrote, “Using different ways to engage us because many people were not comfortable with sharing their cameras.” Another student wrote, “I thought [my instructor] did a good job allowing us to be a part of the class without forcing any of us to speak or turn on our videos when we were not comfortable.”

By not requiring camera use, it is inevitable that at some point one or more students will turn off their video cameras, making it more challenging to identify who is speaking or even present. In this case, we have found it to be helpful to ask students to properly format their name as it appears to others in the videoconferencing program. The name displayed in Zoom, for example, is sometimes a student's obscure university ID, merely their initials, or their legal name instead of their preferred name. We also recommend that students be encouraged to add a static image of their face that will appear when their video is turned off. Furthermore, instructors could set a good example by following these suggestions themselves.

### 4.3 Explicitly encourage camera use, explain why you are doing so, and establish the norm

Instead of requiring video cameras to be turned on, we recommend encouraging it while fostering an inclusive classroom environment. We suspect that some students will not need much encouragement, especially in an age of social distancing to fight the pandemic. For example, one student wrote, “[My instructor] had her camera on [and it] was nice to see another face during times like this [sic].” However, others may need more encouragement.

An early step in encouraging students to use their cameras is to explicitly request it. One of our students wrote, “…if asked to turn my camera on I would have,” and another student indicated that they left their camera off because that was the default setting when connecting with Zoom. We plan to include our camera use policies in the syllabus and have instructors communicate explicit encouragement of camera use on the first day of class, repeating later in the semester if necessary. In addition to communicating when cameras are encouraged to be on, it is also important to explicitly communicate when it would be appropriate to turn them off (and separately, the microphone), like when being interrupted by a family member or trying to improve a weak internet connection.

We also plan to explain to our students why we encourage camera usage, as such transparency should build student buy‐in (Seidel & Tanner, 2013). For example, our instructors could mention some of the many benefits for camera use in remote classes, as reviewed in the introduction section, such as the value of nonverbal cues in communication (Miller, 1988), improved instructor effectiveness (Mottet, 2000), and building instructor–student and student–student relationships (Falloon, 2011; Mottet, 2000). We also think our students would like to know that when we asked past students for suggestions for improving the online Zoom lab meetings, several suggested that cameras be turned on more often because it made class feel “more interactive” and “more like a normal class,” for example. Similar sentiments were reported by students in chemistry classes (Kalman et al., 2020).

By explicitly encouraging camera usage and explaining why we are doing so from the first day of class, we plan to establish video sharing as the classroom norm. Our survey revealed how powerful setting the norm can be. The most frequent “Other” reason students provided for not having their video on was some form of if not being the norm set in the online classroom. For example, “no one else had it on so I shut mine off as well,” “[our class] kept ours off,” and “everyone else had theirs off and I felt awkward having mine on.” This suggests that unspoken social norms are at play and that some portion of students experience the social pressure to follow what their classmates are doing. Classroom interaction norms implicitly set by student peers have been found to significantly shape participation, and it has been suggested that instructors have the power to shape such norms (Fassinger, 1995, 1996).

We believe that the focus theory of normative conduct (Cialdini et al., 1990, 1991) may help to explain our observations and form potential solutions. Applying the theory, students not turning on their cameras because of the perception that that is how most others *are* behaving would be a “descriptive norm.” The more students that the instructor can encourage to turn on their cameras, the greater the student perception that having cameras on is the (descriptive) norm and the more likely students will comply. Another type of norm described by the theory is an “injunctive norm,” which guides a student's behavior based on the perception of how most others *ought* to behave, that is, how most others would approve or disapprove of the behavior. Injunctive norms work even when most are not behaving as desired. Instructors can help set the injunctive norm by explicitly stating that turning cameras on is valued by the instructor and student peers. Theory also posits that a student's actions are more likely to conform to the type of norm that is salient, or in focus, at the time of behavior, so it is ideal if these norms are aligned. To avoid misalignment, we suggest that on the first day of class instructors do not describe the issue of cameras being turned off as unfortunately frequent while they encourage cameras to be on as this would set up contradictory descriptive and injunctive norms. Moreover, aligning norm types makes persuasive messages more likely to be effective (Cialdini, 2003).

We would also like to note that when influencing the injunctive norm, an instructor must be careful to not create the perception that having a camera off is disapproved so much as having a camera on is approved. Part of the classroom norms should be that it is okay to choose not to have your camera on (Stanford University, 2020). An analogy to this would be the injunctive norm that stopping to make a donation (e.g., at a Salvation Army charity kettle) is a behavior that most would approve of, but continuing to walk past is a behavior that most would not likely disapprove of.

Instructors can seek to set the norm of having cameras turned on early on so that students who are willing and able to turn their video on are more comfortable doing so. For example, on the first day of class instructors can devote time for students to introduce themselves to the rest of the class with icebreaker activities like show‐and‐tell. Subsequent classes could begin by checking‐in with each student, asking them to at least briefly turn on their cameras if they are comfortable doing so, and asking fun short questions like “what was your favorite cartoon growing up?” In larger classes where this is not practical, students could dress according to rotating themes like “funny hat day” or “show your school pride day.” While setting the norms for the class, we will be mindful that no student should be made to feel uncomfortable for choosing to leave their video off. Offering alternative methods for participation and building an inclusive classroom should help with not causing any student to feel excluded.

Setting the norm that camera usage is encouraged should also help to address the most frequently cited reason our students gave for not turning on their camera: concern about their personal appearance. If students anticipate that they will likely want to have their camera on during class, they are probably more likely to prepare their appearance by, for example, brushing their hair and not wearing pajamas. It may be also helpful to tell them that a common tip from people whose careers involve working from home is to maintain a routine that often includes getting dressed and good personal hygiene (Knowlton, 2020; Limón, 2020; Rivkind, 2012). We should also note that the Zoom program currently offers a feature to “Touch up my appearance,” which airbrushes a user's face in real time, although we do not promote its use as we suspect it contributes to the negative effects of unrealistic beauty ideals despite being an obvious manipulation (MacCallum & Widdows, 2018).

A state of public self‐awareness, when individuals focus their attention on aspects of the self that are able to be perceived by others, has been shown to be induced by the presence of an audience or even only a video camera (Davies, 2005; Froming et al., 1982; Govern & Marsch, 2001; Myllyneva & Hietanen, 2016). More similar to videoconferencing, the presence of a camera and seeing one's own video image was shown to heighten public self‐awareness in pairs of strangers that were engaged in video chatting (Miller et al., 2017). It stands to reason that a videoconference like those of a Zoom meeting would also increase public self‐awareness.

Increasing public self‐awareness may increase concern about one's personal appearance as that is one aspect of the self that is visible to the public. The level of concern that a student has for their appearance may be due to a number of different psychological and social factors that are beyond the control of the instructor, including aspects of personality (Johnson et al., 2007), culture, gender, and relationship status (Aune & Aune, 1994). As all these factors cannot possibly be addressed by an instructor, we speculate that if students are expecting to turn on their cameras, they will more likely prepare their appearance in a manner that assuages their individual concerns about being seen. While enhanced public self‐awareness is an antecedent to social anxiety, it does not necessarily lead to it (Fenigstein et al., 1975). Nevertheless, instructors should be aware that cameras may trigger such anxiety in students and so this is another reason not to mandate camera use.

To help maintain the norm of camera use throughout the semester and combat a potential decline in usage (e.g., Tonsmann, 2014), we plan to individually reach out to those students that regularly do not turn on their cameras. We will let them know that we have noticed their camera has been off and to ask whether there is anything we can do to help them feel more comfortable with turning it on. When doing so, we will be mindful of student–teacher power dynamics and seek to avoid pressuring a student into doing something they are not comfortable doing. We will also reiterate that camera use will not affect their grade and remind them of the alternative ways to participate in class and to communicate with instructors. Reaching out to students creates the opportunity to make them feel more welcomed and to gain a better understanding of the challenges they face while learning remotely. Knowledge of these challenges may allow us to provide solutions or direct students to helpful resources.

### 4.4 Address potential distractions and give breaks to help maintain attention

While a sizeable number of our students selected concerns about distracting their classmates and their instructor as reasons for not leaving on their video during class, we suggest ways to minimize these distractions while encouraging general camera use.

Students may be assuming that sharing their video feed while the instructor is presenting distracts from the instructor and is thus disrespectful. For example, one student wrote, “I left my video off when [my instructor] would present his slides out of respect so that all the attention would be on him.” Instructors who actually prefer students’ cameras to be on may not realize that some students assume their preference to be the opposite. Obviously, explicitly communicating camera polices that include when it is inappropriate to have cameras on would help.

If students find themselves distracted by the video feeds of their classmates while the instructor is presenting, they can likely alter their personal viewing settings in the videoconferencing program being used. For example, Zoom has a “speaker view” which automatically focuses on the person speaking and an option to pin the instructor's video so that it remains in focus (for an instructional video see Finio, 2020). If, as the results of our survey suggest, some students may be preoccupied by the thought that their classmates are “looking at them the whole time,” it may be comforting to briefly inform students about the “spotlight effect,” which is the tendency of people to overestimate how much they are watched and evaluated by others (Gilovich & Savitsky, 1999). If students are distracted by their own video feed, which is consistent with the findings that mirrors have been shown to increase self‐awareness (Froming et al., 1982; Govern & Marsch, 2001), they should look to see if the videoconferencing software being used has an option to hide it, as Zoom does (Zoom, 2020). Alternatively, students can place a sticky note on the portion of their screen that shows their own video.

The second most frequent reason given for not turning on their camera was concern about other people being seen in the background. There are several reasons why this may be concerning to students such as protecting the privacy of their family or roommates. Regardless of why, students may be distracted by the possibility of someone else coming into view. Similarly, students were concerned about their physical location being seen behind them. Students can be provided suggestions to deal with these concerns, including communicating with roommates and family members about when they have class and ask not to be disturbed, finding a private area of their home where they can connect with minimal distractions (Basile & Beauregard, 2016), staging their background to look more presentable, using a virtual background in the videoconferencing software if their computer allows it, or simply sitting with their back against a wall.

However, we must be mindful that not everyone will have access to a private space, as one of our students pointed out, “It has been difficult to stay focused for long periods of time because there is a lot of commotion in my house, and I have nowhere quiet to go work.” Furthermore, our results suggest that URM students may be disproportionately more concerned about their physical location and other people being seen behind them than non‐URM students. Again, we recommend against requiring cameras be turned on and instead to look for other ways to promote inclusion.

To help maintain attention, we also encourage giving students breaks, especially with longer classes. Students can use that time to go to the bathroom, get something to eat, and relax while fighting off “Zoom fatigue” (Jiang, 2020; Morris, 2020). They will then also be less likely to turn off their video and take a break during class anyway. When students return from break, it is potentially a good time to encourage cameras to be turned on again by facilitating an activity that makes use of them.

### 4.5 Use active learning techniques to keep students engaged and promote equity

Several of our colleagues at Cornell and other institutions have expressed concern that students sometimes leave their video turned off in order to not be seen being inattentive or doing other things. Indeed, this does seem possible based on our students having selected as reasons for leaving their video off: not wanting to be seen not paying attention, walking away from the computer, and doing other things on their computer. Students may be bored or falling victim to the perils of multitasking (Klemm, 2016; Mokhtari et al., 2015). To capture student attention, while also improving student learning, we suggest regularly employing active learning techniques which have been shown to increase persistence in STEM, improve academic performance, and lower failure rates (Braxton et al., 2008; Freeman et al., 2014). Furthermore, active learning was found to narrow achievement gaps between students from overrepresented and underrepresented groups by improving examination scores and passing rates for all student groups, but disproportionately so for those from underrepresented minorities (Theobald et al., 2020). However, it should be noted that active learning does not guaranty equity and instructors should be vigilant of inequity due to a number of factors including small group dynamics (e.g., Reinholz & Shah, 2018) or potential hidden biases (e.g., Ernest et al., 2019).

By establishing engaging activities such as polling or typing into the chat window as a regular part of class, students should be less tempted to do other things during class and thus less likely to turn off their video in order to conceal themselves. Using the think‐pair‐share/listen‐think‐pair‐share technique (Lyman, 1981; Tanner, 2013) along with Zoom's breakout room feature may be a particularly helpful in this regard. Based on feedback from our instructors and students, some students seem to be more willing to turn on their video when the class is split up into smaller breakout rooms within Zoom. This pattern has also been reported by our colleagues at Cornell and other universities. We also recommend incentivizing students with a participation grade while promoting equity by providing multiple ways to participate in active learning that does not require sharing video, mentioned above. Keeping students actively engaged may also have the added bonus of decreasing the likelihood that they feel like everyone is looking at them the whole time.

### 4.6 Survey your students to understand their challenges

Our end‐of‐semester survey has given us information we have used to plan for the fall semester as the COVID‐19 pandemic continues. We have shared our data‐driven recommendations for encouraging camera use here but caution other instructors to consider that the challenges facing their student population may differ and require different solutions.

In addition to an end‐of‐semester survey, we surveyed our students multiple times to better understand the challenges they were facing in the switch to emergency remote instruction, and what we learned influenced our teaching practices. For example, we were initially concerned about the “digital divide” (Cullen, 2001) in that many of our students would not have access to a reliable internet connection or to a computer with a working webcam and microphone. However, only a small percentage of students indicated these problems (webcam: 2%; internet: 5%; *N* = 301) in a survey we gave during a break in classes before remote instruction began. This partly informed our conclusion that it was not unreasonable to hold live synchronous class meetings that encouraged the use of webcams while also offering asynchronous learning opportunities to those that could not attend. Those students without reliable access to the necessary technologies were also referred to university resources set up in response to the pandemic.

## 5 CONCLUSIONS

Our end‐of‐semester survey of students revealed insights into why students might have chosen not to turn on their video cameras during online synchronous class meetings held via Zoom. This information has helped us to develop a plan for encouraging—but not requiring—camera use as we continue remote instruction during the COVID‐19 pandemic. While the demographics and needs of students vary by course and institution, we believe that these strategies are likely to be helpful to many instructors. Our data‐driven plan also provides a model for other instructors to gather information about their own student population to develop more tailored strategies that also promote equity and inclusion.

## CONFLICT OF INTEREST

None declared.

## AUTHOR CONTRIBUTIONS

**Frank R. Castelli:** Conceptualization (lead); data curation (lead); formal analysis (lead); investigation (equal); methodology (lead); visualization (lead); writing – original draft (lead); writing – review and editing (equal). **Mark A. Sarvary:** Funding acquisition (lead); investigation (equal); methodology (supporting); writing – review and editing (equal).

## Data Availability

In order to best protect student privacy, we decline to share data beyond what is reported in the manuscript.
